# Violence victimization and perpetration within the caregiver-patient relationship in schizophrenia: A cross-sectional study in Tunisia

**DOI:** 10.1371/journal.pone.0323312

**Published:** 2025-05-30

**Authors:** Feten Fekih-Romdhane, Bochra Nourhène Saguem, Manel Stambouli, Souheil Hallit, Majda Cheour

**Affiliations:** 1 The Tunisian Center of Early Intervention in Psychosis, Department of Psychiatry “Ibn Omrane”, Razi hospital, Manouba, Tunisia; 2 Tunis El Manar University, Faculty of Medicine of Tunis, Tunis, Tunisia; 3 Research Laboratory LR12ES04, Farhat Hached Hospital, Department of Psychiatry, University of Sousse, Sousse, Tunisia; 4 School of Medicine and Medical Sciences, Holy Spirit University of Kaslik, Lebanon; 5 Department of Psychology, College of Humanities, Effat University, Jeddah, Saudi Arabia; 6 Applied Science Research Center, Applied Science Private University, Amman, Jordan; Istanbul Bakirkoy Prof Dr Mazhar Osman Ruh Sagligi ve Sinir Hastaliklari Egitim ve Arastirma Hastanesi, TÜRKIYE

## Abstract

**Background:**

Family caregivers represent an integral part of the mental health care system in Tunisia, as well as in most of the low-to-middle-income countries and collectivist cultural backgrounds (i.e., communities that prioritizes the group over the individual). However, their task is burdensome, often associated with both experienced and perpetrated violence toward the patient. We aimed to shed light on the caregiver-patient relationship by investigating the prevalence and correlates of both violence victimization and violence perpetration in a Tunisian sample of caregivers of patients with schizophrenia.

**Method:**

A paper-and-pencil self-administered questionnaire was used to collect data from participants. Caregivers (N = 110, aged 39.7 ± 12.5 years, 63.6% females) were asked questions about their experience of violence perpetration and victimization involving their relative with schizophrenia in the past 12 months. The Depression Anxiety and Stress Scales (DASS-21) and the abridged version of the Zarit Burden Interview (ZBI) were administered to all participants.

**Results:**

Verbal violence was the most reported type of violence victimization (35.5%), followed by threat (25.5%), and physical violence (25.5%). In addition, 54.5% of caregivers disclosed having perpetrated verbal violence at least once against their ill relative. The most endorsed causes of violence victimization were symptoms of illness (57.3%) and refusal to adhere to treatment (49.1%). Multivariable analysis (logistic regression) revealed that caregivers’ levels of burden remained significantly associated with violence victimization occurrence (OR = 1.48; 95% CI 1.05; 2.09; p = .026), while having another person in charge of caring represented a significant factor associated with perpetration of any form of violence against patients (OR = .17; 95% CI.05;.62; p = .007).

**Conclusion:**

Although preliminary and based on cross-sectional data and a relatively small sample size, our findings draw attention to the high prevalence of both violence perpetration and victimization within the caregiver-patient relationship in schizophrenia. Findings also identified caregivers’ burden and having another person in charge of caring as factors significantly associated with occurrence of any violence victimization and violence perpetration, respectively. These factors are potentially malleable, and may be helpful in targeting an at-risk population and developing appropriate prevention strategies.

## Introduction

Schizophrenia is a chronic and highly burdensome psychiatric illness associated with delusional symptoms, negative symptoms, impaired functioning, altered quality of life, social exclusion, and stigma [1,2]. In dealing with such a challenging illness, caregivers of patients with schizophrenia play a significant role not only through providing informal care and support to patients but also through guaranteeing and managing their medical care [3]. Though they may constitute a heterogeneous group, caregivers of patients with schizophrenia are most commonly their close relatives [4]. Caregivers’ role in improving patients’ outcomes and functioning has been undeniable [5]. Patients with schizophrenia who had a good caregiver support showed significantly better outcomes compared to patients who had not, including significantly lower numbers and durations of hospitalizations, significantly lower relapse rates, significantly lower mortality rates, and improved compliance to treatments [6–8]. Despite these favorable outcomes, caregivers’ task is associated with psychological distress and considerable burden of care [3,4]. Additionally, caregivers of patients with schizophrenia may be exposed to verbal and physical violence from the patients, and also perpetrate violence to them [4,9]. The risk of manifesting violent behaviors is increased in patients with schizophrenia [10]. Approximately half of the patients’ victims are their relatives [11]. In another hand, patients with schizophrenia are at an increased risk of being victims of violence compared to the general population [12].

### Risk factors for violence in the caregiver-patient dyad in schizophrenia

Aggression refers to an observable behavior, that is carried out intentionally, and aiming to harm in different forms (such as hurt feelings, physical injury, or damaged social relationship) [13]. Violence can be regarded as “a subset” and an “extreme form” of aggression [14]. Indeed, it is commonly accepted that both violence and aggression lie on a continuum of severity, with violence (e.g., homicide) being at the highest end of the continuum, while relatively minor acts of aggression (e.g., pushing) exist at the lowest end of the continuum [14]. Potential risk factors for violence are important to be determined because when addressed or eliminated, they can prevent the likelihood of its occurrence. Victimization and perpetration of violence are both multifactorial, resulting from complex interactions between the psychiatric illness, the caregiver-patient relationship, as well as daily interactions and circumstances [15]. Several risk factors for violence related to schizophrenia have consistently been identified in previous meta-analytic findings, including younger age, male gender, lower levels of education, a forensic history, duration of untreated psychosis, substance use, severity of psychotic symptoms, hostile behavior, poor impulse control, non-adherence with medication, and involuntary treatment (e.g., forced medication, involuntary psychiatric hospitalization, physical restraint) [16–18]. Although most of these risk factors appear to be universal, other factors are rather culturally specific [19,20]. One of the culturally specific risk factors for violence is Trihexyphenidyl, which is an anti-Parkinson and anticholinergic drug commonly prescribed in patients with schizophrenia in Tunisia [21]. Trihexyphenidyl has been shown to be potentially misused by patients in the Tunisian context [21–23], thus leading to increased risk of violence. Other hypothesized factors specific to the Tunisian context that might be associated with violence in schizophrenia are first-generation antipsychotics (FGAs) intake and the concurrent use of two (or more) antipsychotic medications [24]. This can be explained by the fact that therapeutic options are limited in Tunisian public health care settings, with only a few oral (and no long‐acting injectable) second-generation antipsychotics (SGAs) available. As such, severe or resistant psychotic symptoms, as well as high levels of violence/aggressiveness, may lead treating psychiatrists to resort to a switch to FGAs or even the use of antipsychotic polypharmacy combining FGAs with another SGAs. FGAs remain highly prescribed in Tunisia [25]. For instance, a Tunisian study found that 80.6% and 52.8% of individuals with severe mental illness (69.4% with schizophrenia) who committed homicide were under oral and long-acting FGAs, respectively. Overall, these data suggest that it is crucial to further understand and consider cross-countries and cross-cultural heterogeneity when investigating factors associated with violence in schizophrenia.

### Caregiver-patient dynamics in schizophrenia in the Tunisian context

To the best of our knowledge, no previous studies have addressed violence in the caregiver-patient dyad in schizophrenia in an Arab developing country, with the vast majority of the existing literature having mainly emerged from the Western world [26,27]. However, aggression and violence, both experienced and perpetrated (in particular within the family nucleus), is a complex phenomenon that is subject to cultural variations in terms of prevalence, nature, and outcomes [28–30]. Based on the individualism-collectivism dimension, Arab societies and cultures are classified as collectivist [31], where family cohesion, respect for parental authority, and interdependent ties are still strongly emphasized [32]. However, violent and aggressive behaviors have become increasingly common over the last decades in Arab countries including within families [33–35] because of rapid social changes, increased rates of substance use, unprecedented economic crises, as well as other macro-level determinants [27]. To date, a dearth of literature exists on aggression and violence risk involving Tunisian patients with schizophrenia and/or their caregivers. A study by Ellouze et al. reported an estimated prevalence of violence of 26.08% in patients diagnosed with schizophrenia [36]. Around three out of four Tunisian family caregivers of patients with psychosis experienced moderate to severe levels of aggression from their ill relatives [37]. Another study showed that the number of homicides committed by people with mental illness increased 1.3 times since 2011 in Tunisia, with the crime scene being family home in 51.2% of cases, and the victim being a parent/spouse/sibling in 41.5% of cases [38]. At the same time, offense recidivism in Tunisian patients with schizophrenia has been associated with residing without a family after hospital discharge [24]. These alarmingly high estimates prompt a deeper examination of this phenomenon and its related factors. Such research is particularly relevant in Tunisia, a developing and middle-income country where psychiatric healthcare is mainly hospital-based and secondary care, with no community-based care or rehabilitation and recovery services available for patients with serious mental illnesses. As such, treating psychiatrists mostly rely on family caregivers as a fundamental and integral part of the management of these diseases, hence the major importance of ensuring the integrity of the caregiving relationship in schizophrenia.

### The present study

The present study was motivated by a lack of research on violence within the dyad caregiver-patient with schizophrenia. Most of the reported studies on the topic have addressed violence committed by the patients towards their caregivers rather than violence perpetrated towards them [39]. Besides, there is a lack of studies addressing this issue in some parts of the world, such as North Africa and the Arab world. This study aimed to shed light on the relationship caregiver-patient with schizophrenia by investigating the prevalence and correlates of both violence victimization and violence perpetration among a Tunisian sample of caregivers of patients with schizophrenia. It is hypothesized that: (1) a high proportion of caregivers would exhibit high rates of violence victimization and violence perpetration towards their ill relatives, and (2) some caregivers’ characteristics, such as burden and psychological distress, would be associated with perpetration and victimization of violence between caregivers and their relatives.

## Methods

### Ethics approval and consent to participate

The protocol of the study was approved by the ethics committee of the Razi Psychiatric Hospital (reference number: ECRPH-2022–0055; approval date: 04/04/2022). Informed consent was obtained from all subjects; submitting the form online was considered equivalent to obtaining a written informed consent.

### Participants and procedure

We carried-out a cross-sectional study among caregivers of patients with schizophrenia during the period from June to August 2022. Patients who attended the department of psychiatry with their formal caregivers for a consult were identified and approached by a study team member, then screened for eligibility and informed about the study while waiting for their consult. Patients’ eligibility criteria consisted of (1) age 18 years and older, (2) meeting diagnosis of schizophrenia, confirmed by two independent expert psychiatrists according the DSM-5 criteria, for at least one year, and (3) meeting criteria for clinical remission for at least three months before the survey. We clearly explained to patients and their caregivers the nature and goals of the study. Informed consent was obtained from all participants prior to collecting data. The patient’s refusal to participate led to the non-enrollment of the caregiver.

If interested and willing to participate, only one caregiver per eligible patient was interviewed. Each participating caregiver was led to a private room by an investigator and invited to complete an anonymous survey (while patients were attending their regular outpatient appointments). Caregivers should meet the following criteria to be enrolled into the study: (1) age 18 years and over, (2) being the main care provider for the patient for at least one year (i.e., providing informal, unpaid care and support including supervising medications, accompanying patients to the hospital, continuing contact with the hospital staff, assistance with daily needs, emotional comfort and support) [40], and (3) being able to fill out the questionnaire by themselves (e.g., being able to read Arabic). Overall, 110 caregivers of patients with schizophrenia (mean age: 50.8 ± 4.0 years) were included in the present study.

### Minimum sample size

The G-power software estimated a minimum sample of 42 patients, based on the statistical test multiple regression (R2 deviation from zero) taking a small f2 value of 0.02, a 5% alpha risk of error, a power of 80% and 6 predictors to be entered in the multivariable model.

### Measures

A paper-and-pencil self-administered questionnaire was used to collect data from caregivers, while patients’ clinical information was obtained from medical records. The questionnaire was divided into three sections. The first section contained items regarding patient- and caregiver-related information. For patients, sociodemographic information (age, gender, educational level, occupation, marital status, economic status, residency), clinical data (history of suicide attempts, substance use, Age at illness onset, duration of disease, duration of untreated psychosis (years), number and ways of hospitalizations, treatment characteristics) were gathered. Educational level was categorized into primary (i.e., elementary school), secondary (i.e., high-school), and tertiary (i.e., This refers, in the Tunisian education system, to university education pursued beyond the high school level). Self-perceived economic status was categorized subjectively by respondents into three groups: low, average and high. For caregivers, in addition to sociodemographic data (age, gender, education, occupation, marital status, economic status), relation with patient, personal psychiatric history, and having another person in charge of caring were also collected.

In the second section (attached as an Appendix), caregivers were asked questions about their experience of violence perpetration and victimization involving their relative with schizophrenia in the past 12 months. These questions were closely adapted from a previous Chinese study on the same topic [41]. They were based on the definition of violence as involving both psychological (verbal or non-verbal) and physical methods that may result in mental/emotional harm to others, disability, destruction, or even death [42]. Both perpetration and victimization of violence between respondents and their relatives were classified into four types: (1) “language violence” (the use of abusive, insulting or discriminatory language, which may cause psychological harm), (2) “threat” (verbal or behavioral threats, such as intimidating communication, confrontation, stomping, slapping the table), (3) “physical violence” (the use of physical force to inflict intentional harm or injury, including hitting, slapping, kicking, punching, pushing, biting, hair pulling, scratching), and (4) “violence against property” (acts that damage properties, such as furniture, money, cars, without the owner’s permission). Caregivers were asked to indicate the frequency of each type of violence. Then, two dichotomous violence outcomes were created for analyses: any violence victimization (at least one type of violence victimization was reported) versus no violence victimization, and any violence perpetration (at least one type of violence perpetration occurred) versus no violence perpetration. Beyond frequency, caregivers were also asked to specify, among the following options, the causes of the violence perpetrated and suffered: symptoms of illness, refusal to adhere to treatment, refusal of hospitalization, limitation of patients’ activities and/or liberty, treating the patient unfairly due to their illness, drug reaction, negative events, others.

The third section contained two measures, i.e., the Depression Anxiety and Stress Scales (DASS-21) and the abridged version of the Zarit Burden Interview (ZBI), assessing psychological distress and caregiving burden, respectively.

The DASS-21 [43] involves three scales of seven items, each one reflecting the severity of depression, anxiety and stress symptoms. The scale contains 21 items rated on a four-point Likert scale from 0 (did not apply to me at all) to 3 (applied to me very much, or most of the time). The DASS-21 has showed good psychometric quality in different clinical and non-clinical samples [44]. The Arabic version of the scale was used [45], and showed good reliability in the current sample (Cronbach’s alpha for total score = 0.89).

The ZBI [46] is a 12-item measure rated on a five-point Likert scale ranging from 0 (never) to 4 (almost always). Total scores range from 0 to 48, with high scores indicating greater sense of caregiving burden. The Arabic version used in the preset study showed adequate psychometric properties [47], and yielded a Cronbach alpha value of 0.87 in our sample.

### Statistical analyses

We analyzed data using SPSS, v. 26. The Shapiro–Wilks test was used to compare the distribution of continuous variables to a normal distribution. Since our variables were normally distributed, means and standard deviations were calculated for quantitative variables. Univariate analyses exploring associations between study variables and violence victimization/perpetration (Yes/No) were performed using Student t test for quantitative variables and Chi-square test for qualitative variables. Then, a logistic regression of factors associated with both Violence Victimization and Violence Perpetration has been performed, taking into account factors that showed a p < .25 in the bivariate analysis as independent variables. The significance level was set at.05 in all statistical tests.

## Results

The majority of caregivers were females (63.6%), and consisted of patients’ parents (50.9%). Other caregiver-related and patient-related information are presented in tables 1 and 2, respectively. Verbal violence was the most reported type of violence victimization (35.5% reported having suffered this form of violence more than three times during the last 12 months), followed by threat (25.5%), and physical violence (25.5%). In addition, 54.5% of caregivers disclosed having perpetrated verbal violence at least once against their ill relative, while 10.9% admitted having committed at least three acts of physical violence during the last year (Table 3). As shown in Table 4, the most endorsed causes of violence victimization were symptoms of illness (57.3%), followed by refusal to adhere to treatment (49.1%), drug reaction (23.6%), and negative events; while the most reported causes of violence perpetration were refusal to adhere to treatment (42.7%), Symptoms of illness (37.3%), and limitation of patients’ activities and/or liberty (32.7%).

**Table 1:** Characteristics of caregivers significantly associated with violence victimization and/or perpetration.

	Characteristics of caregivers (N = 110)	Violence victimization		*t* or χ^2^	*p*	Violence perpetration		*t* or χ^2^	*p*
	n (%) or mean±SD	No (n = 17)	Yes (n = 93)			No (n = 37)	Yes (n = 73)		
**Gender**				1.43	.282			7.33	**.007**
Male	40 (100%)	4 (10.0%)	36 (90.0%)			7 (17.5%)	33 (82.5%)		
Female	70 (100%)	13 (18.6%)	57 (81.4%)			30 (42.9%)	40 (57.1%)		
**Education level**				8.15	**.017**			2.27	.322
Primary	54 (100%)	6 (11.1%)	48 (88.9%)			17 (31.5%)	37 (68.5%)		
Secondary	40 (100%)	11 (27.5%)	29 (72.5%)			12 (30.0%)	28 (70.0%)		
Tertiary	16 (100%)	0 (0%)	16 (100%)			8 (50.0%)	8 (50.0%)		
**Occupation**				19.21	**<.001**			.01	.941
Unemployed	50 (100%)	16 (32.0%)	34 (68.0%)			17 (34.0%)	33 (66.0%)		
Employed	60 (100%)	1 (1.7%)	59 (98.3%)			20 (33.3%)	40 (66.7%)		
**Relation with patient**				.51	.918			1.93	.588
Spouse	21	4 (%)	17 (%)			8 (%)	13 (%)		
Sibling	25	3 (%)	22 (%)			8 (%)	17 (%)		
Child	8	1 (%)	7 (%)			1 (%)	7 (%)		
Parent	56 (%)	9 (%)	47 (%)			20 (%)	36 (%)		
**Having another person in charge of caring**				5.60	**.027**			13.46	**<.001**
No	41 (100%)	2 (4.9%)	39 (95.1%)			5 (12.2%)	36 (87.8%)		
Yes	69 (100%)	15 (21.7%)	54 (78.3%)			32 (46.4%)	37 (53.6%)		
**Burden**	9.0 ± 7.5	3.76 ± 1.64	9.98 ± 7.74	-6.93	**<.001**	8.5 ± 7.8	9.26 ± 7.39	-.48	.636
**Depression**	12.8 ± 6.9	6.82 ± 2.46	13.94 ± 6.92	-7.63	**<.001**	7.9 ± 4.6	15.3 ± 6.6	-6.79	**<.001**
**Anxiety**	11.6 ± 7.4	5.29 ± 2.34	12.82 ± 7.47	-7.83	**<.001**	7.6 ± 5.4	13.7 ± 7.5	-4.85	**<.001**
**Stress**	12.7 ± 7.4	6.35 ± 2.94	13.83 ± 7.37	-7.16	**<.001**	7.5 ± 4.4	15.3 ± 7.2	-7.00	**<.001**

Note: SD: standard deviation; Bold values: significant at p < .05.

**Table 2:** Patients’ sociodemographic and clinical characteristics significantly associated with violence victimization and/or perpetration among primary caregivers.

	Characteristics of patients (N = 110)	Violence victimization		*t* or χ^2^	*p*	Violence perpetration		*t* or χ^2^	*p*
	n (%) or mean±SD	No (n = 17)	Yes (n = 93)			No (n = 37)	Yes (n = 73)		
**Education level**				7.34	**.026**			1.91	.384
Primary	54 (100%)	4 (7.4%)	50 (92.6%)			19 (35.2%)	35 (64.8%)		
Secondary	38 (100%)	7 (18.4%)	31 (81.6%)			10 (26.3%)	28 (73.7%)		
Tertiary	18 (100%)	6 (33.3%)	12 (66.7%)			8 (44.4%)	10 (55.6%)		
**Self-perceived economic Status**				1.08	.409			4.12	**.042**
Low	38 (100%)	4 (10.5%)	34 (89.5%)			8 (21.1%)	30 (78.9%)		
Average/ High	72 (100%)	13 (18.1%)	59 (81.9%)			29 (40.3%)	43 (59.7%)		
**Residency**				7.07	**.014**			2.88	.104
Rural	45 (100%)	2 (4.4%)	43 (95.6%)			11 (24.4%)	34 (75.6%)		
Urban	65 (100%)	15 (23.1%)	50 (76.9%)			26 (40.0%)	39 (60.0%)		
**Personal medical history**				5.07	**.046**			.92	.490
No	100 (100%)	13 (13.0%)	87 (87.0%)			35 (35.0%)	65 (65.0%)		
Yes	10 (100%)	4 (40.0%)	6 (60.0%)			2 (20.0%)	8 (80.0%)		
**Tobacco use**				19.55	**<.001**			5.90	**.015**
No	34 (100%)	13 (38.2%)	21 (61.8%)			17 (50.0%)	17 (50.0%)		
Yes	76 (100%)	4 (5.3%)	72 (94.7%)			20 (26.3%)	56 (73.7%)		
**Alcohol use**				12.17	**<.001**			6.93	**.014**
Never	61 (100%)	16 (26.2%)	45 (73.8%)			27 (44.3%)	34 (55.7%)		
Occasionally	49 (100%)	1 (2.0%)	48 (98.0%)			10 (20.4%)	39 (79.6%)		
**Lifetime number of hospitalizations**	3.5 ± 2.8	1.7 ± 1.5	3.88 ± 2.83	-3.08	**.003**	3.5 ± 3.5	3.5 ± 2.3	-.01	.990
**Lifetime number of involuntary admission**	3.5 ± 2.8	1.7 ± 1.5	3.85 ± 2.87	-2.99	**.003**	3.8 ± 3.9	3.4 ± 2.0	.70	.484
**Antipsychotics class**				14.18	**<.001**			.117	.840
Second-generation	51 (100%)	15 (29.4%)	36 (70.6%)			18 (35.3%)	33 (64.7%)		
First-generation	59 (100%)	2 (3.4%)	57 (96.6%)			19 (32.2%)	40 (67.8%)		
**Long-acting injectable antipsychotics**				.05	.776			.90	.375
No	30 (100%)	5 (16.7%)	25 (83.3%)			8 (26.7%)	22 (73.3%)		
Yes	80 (100%)	12 (15%)	68 (85%)			29 (36.2%)	51 (63.7%)		
**Trihexyphenidyl**				3.93	.069			10.91	**.001**
No	18 (100%)	0 (0%)	18 (100%)			0 (0%)	18 (100%)		
Yes	92 (100%)	17 (18.5%)	75 (81.5%)			37 (40.2%)	55 (59.8%)		
**Benzodiazepines**				1.67	.244			1.32	.256
No	95 (100%)	13 (13.7%)	82 (86.3%)			30 (31.6%)	65 (68.4%)		
Yes	15 (100%)	4 (26.7%)	11 (73.3%)			7 (46.7%)	8 (53.3%)		
**Mood stabilizers**				.65	.554			.90	.375
No	80 (100%)	11 (13.7%)	69 (86.2%)			29 (36.2%)	51 (63.7%)		
Yes	30 (100%)	6 (20%)	24 (80%)			9 (30%)	22 (73.3%)		

Note: SD: standard deviation; Bold values: significant at p < .05.

**Table 3:** Frequency of each type of violence vctimization and perpetration among primary caregivers over the last 12 months.

Frequency	Victimization				Perpetration			
	Verbal	Physical	Threat	Against objects	Verbal	Physical	Threat	Against objects
**0**	27.3%	42.7%	54.5%	82%	45.5%	63.6%	70.9%	79.1%
**1**	14.5%	12.7%	12.7%	3%	8.2%	16.4%	16.4%	20.9
**2**	22.7%	25.5%	7.3%	9%	9.1%	9.1%	2.7%	0
**≥3**	35.5%	19.1%	25.5%	6%	37.2%	10.9%	10.0%	0

**Table 4:** Number and percentage of caregivers who considered the factors listed as reasons for violence victimization or perpetration toward the patient.

	Causes of Violence Victimization (N = 110)	Causes of Violence Perpetration (N = 110)
**Symptoms of illness**	63 (57.3%)	41 (37.3%)
**Refusal to adhere to treatment**	54 (49.1%)	47 (42.7%)
**Refusal of hospitalization**	22 (20.0%)	32 (29.1%)
**Limitation of patients’ activities and/or liberty**	20 (18.2%)	36 (32.7%)
**Treating the patient unfairly due to their illness**	21 (19.1%)	19 (17.3%)
**Drug reaction**	26 (23.6%)	13 (11.8%)
**Negative events**	25 (22.7%)	14 (12.7%)

Bivariate analysis showed that lower caregivers’ educational level (p = .017), unemployment (p < .001), other person in charge (p = .027), burden levels (p < .001), depression (p < .001), anxiety (p < .001) and stress (p < .001) symptoms are positively associated with violence victimization occurrence; while being male caregiver (p = .007), having another person in charge of caring (p < .001) and higher levels of depression (p < .001), anxiety (p < .001), and stress (p < .001) were associated with more violence perpetration. On the other hand, lower patients’ educational level (p = .026), being from rural area (p = .014), personal organic history (p = .046), tobacco use (p < .001), alcohol use (p < .001), lifetime number of hospitalizations and involuntary admission (p = .003), and taking FGAs (p < .001) were associated with more violence victimization. Lower patients’ economic status (p = .042), tobacco (p = .015) and alcohol use (p = .014) as well as taking Trihexyphenidyl (p = .001) were significantly and positively associated with violence perpetration by caregivers against their relatives with schizophrenia. Other bivariate analyses results are displayed in Supplementary tables 1 and 2.

Multivariable analysis (logistic regression) revealed that caregivers’ levels of burden remained significantly associated with violence victimization occurrence (OR = 1.48; 95% CI 1.05; 2.09; p = .026), while having another person in charge of caring represented a significant factor associated with perpetration of any form of violence against patients (OR = .17; 95% CI.05;.62; p = .007) (Table 5).

**Table 5:** Multivariable analysis (logistic regression) of factors associated with Violence Victimization and Violence Perpetration among primary caregivers (N = 110).

	*p*	OR	95% CI
**a. Logistic regression (using the ENTER method) of factors associated with Violence Victimization (Yes/No) (Nagelkerke R** ^**2**^ ** = .454).**			
Burden	***.026***	1.48	1.05; 2.09
Depression	*.957*	.99	.81; 1.22
Anxiety	*.213*	1.15	.93; 1.42
Stress	*.118*	1.16	.96; 1.40
**b. Logistic regression (using the ENTER method) of factors associated with Violence Perpetration (Yes/No) (Nagelkerke R** ^**2**^ ** = .475).**			
Depression	*.113*	1.11	.98; 1.27
Anxiety	*.584*	1.04	.91; 1.19
Stress	*.090*	1.11	.98; 1.25
Having another person in charge of caring (yes vs no*)	***.007***	.17	.05;.62

## Discussion

Caregivers’ role in improving patients’ outcomes and functioning has been undeniable. Nevertheless, their task is burdensome and it is associated with psychological distress, experiencing violence from patients with schizophrenia, and also perpetrating violence to them [4,9]. This study aimed to assess violence victimization and violence perpetration among a Tunisian sample of caregivers of patients with schizophrenia, and to determine their main associated factors. Results revealed that a considerable proportion of the study sample reported violence victimization and violence perpetration towards patients with schizophrenia with a higher proportion of violence victimization.

### Prevalence of violence victimization and violence perpetration among caregivers

Verbal and physical violence were the most frequently reported ones in both of the cases. Threats and violence against objects were most frequently cited by the caregivers as types of violence perpetration. According to prior research, lifetime caregivers’ exposure rates to patients’ violence range from 50 to 60% [4,48]. About one-third of the reported violent incidents has occurred during the preceding year [48]. In a rate comparable to this study result, exposure to moderate to severe levels of aggressive behaviors was reported by 77.4% of the relatives of patients with severe mental illness during the preceding year [49]. Of these, 32.6% stated that they had feared for their lives consequently to the perpetrated violence towards them [49]. In another study including 61 caregivers and relatives to patients with schizophrenia, psychological and physical aggression were reported by 64% and 31% of the sample, respectively [50]. Violence manifested by the patients towards their caregivers generally occurs in their co-residence [15]. Additionally, patients regularly inflict violent behaviors towards their caregivers before their homicide occurs [51]. Despite these alarming reported frequencies of violence victimization among caregivers of patients with schizophrenia, it is noteworthy that they are likely to be underestimated [4]. Indeed, social desirability bias may influence the reported rates of violence [52]. Additionally, manifestation of violent behaviors is considered by family members as a predetermined consequence of the illness and it would quite often be tolerated by them [53]. In line with this, symptoms of the illness were the most frequently reported cause of violence victimization among caregivers in this study. Contrary to violence victimization, data regarding caregivers’ perpetration of violence that involves patients with mental illness are scant [9]. We found that almost two in three caregivers reported having committed any form of violence against their ill relatives, which is consistent with previous findings documenting high rates of violence perpetration among caregivers of patients with psychiatric disorders [39]. Of note, many authors outlined the bidirectional nature of violence manifested between patients with mental illness and their caregivers, especially when they share the same residence [39,54]. Indeed, Labrum et al. found that 55% of the violence manifested since the diagnosis of the mental illness was bidirectional [39]. Additionally, 44% of the violence manifested during the six preceding months was bidirectional too [39].

From a cultural perspective, caring for vulnerable and ill parents is a reflect that Muslims are obedient to God, as stated in one of the Hadiths, “Goodness towards (one’s) parents is the greatest obligatory act” [55]. As such, caring for a sick family member is considered as a heavy cultural “duty”, “responsibility”, “religious obligation” in Arab Muslim societies [55], where the caregiver is expected to accept the new family role given by God without complaint and live it with trust [56]. Family caregiving in Muslim societies is regarded as an opportunity to atone for misdeeds, and gather good deeds that would be rewarding either in this world or in the hereafter [57]. Hence, caregivers keep doing their caregiver duties and responsibilities despite having a devastated life [55]. On the other hand, caregivers reported also feeling “unprepared” for the unforeseen change imposed to them [55], and not adequately guided by the health system on how to care for the ailing family member at home [58], which can lead them to substantial burden and serious consequences, such as maltreatment towards the ill relative. It is of note that family violence is widespread in different Arab countries, considered by some authors as “a veiled epidemic” (for review, see [59]). One of reason is, for example, the divine right parents believe they have to discipline their off-spring. Indeed, misinterpretation and manipulative use of Quran and Hadith can both lead men to commit disciplinary physical violence against their children and even spouses in the name of Islamic teachings, while refusing accountability for their actions [59]. This is supported by our results showing that significantly more male caregivers perpetrated violence against their ill relative. The latter finding can also be explained by the patriarchal structure of Arab societies, where women lack authority and power in the family, and where violence perpetrated by men against women is endorsed by the society itself [60]. These observations, along with the current results further support of Bandura’s social learning theory of aggression [61], which stipulates that an important factor that seems to play a “reinforcer” role in the perpetration of aggressive behaviors is social approval or increased status. According to Bandura [61], individual subcultures, family members, and mass media represent the three major sources of aggressive modeling (i.e., learning the modeled aggressive behavior by observation). Because of all these considerations, violence within the family can be normalized in Arab cultures and societies, and thus be under-studied and under-reported.

### Factors associated with violence directed against caregivers

Among the caregivers’ characteristics, having a lower educational level and a greater sense of caregiving burden were positively associated with violence victimization occurrence. This study’s results expand the literature stipulating a positive association between violence victimization and caregivers’ great sense of burden [4,62]. Caregivers’ burden may be manifested in an objective and a subjective dimensions [63]. Objective burden may include financial burden, effects on physical and mental health of family members, effects on family routine and on family leisure activities. Subjective burden represents the extent to which the feeling of carrying a burden is manifested [63]. Caregivers of patients diagnosed with schizophrenia manifest extensive burden. In a sample of 368 caregivers, mostly parents of these patients, 85.3% and 84.2% showed amounts of objective and subjective burden, respectively [63]. Many factors associated with violence victimization in this study were found to be associated with caregivers’ burden too, including limited educational level, financial burden, and also psychological distress [3,63]. Burden in care has been associated with psychological distress in caregivers [3,64]. Violence victimization has also been associated with fear and psychological distress [4]. Caregivers of patients diagnosed with schizophrenia often display depressive and anxiety symptoms [64]. However, in this study, the greater sense of caregiving burden predicted violence victimization among caregivers upon and beyond psychological distress.

Among the patients’ characteristics, having a lower educational level, more tobacco and alcohol use, a higher lifetime number of hospitalizations (including more involuntary admissions), and being prescribed first-generation antipsychotics were positively associated with violence victimization occurrence among caregivers, whereas the long-acting injectable form showed no significant association with violence risk. Previous studies reported that patients with schizophrenia whose level of education was lower exhibited significantly higher risk of violence in patients [65,66]. Moreover, tobacco use has previously been documented to be associated with violent behavior after illness onset in patients with schizophrenia and related disorders [67]. In addition, alcohol use was consistently recognized as the strongest predictor of physical violence and violent offending in patients with schizophrenia [68]. As for medication, and in line with our results, a large nationwide Swedish study of 74 925 individuals found that the relative risks of all crime outcomes (including violent, drug-related, any criminal arrests and convictions) were substantially decreased in patients who were prescribed second-generation antipsychotics (clozapine, olanzapine, risperidone) compared to those who were prescribed haloperidol [69]. In contrast to our findings, the same study indicated that long-acting injectables were linked to reduced violence risk [69]. This inconsistency could be explained by the fact that only first-generation antipsychotics are available in long-acting injectable form in Tunisia. Finally, other medication, such as mood stabilizers, were not found to be associated with violence behavior perpetrated by patients against their caregivers. Likewise, Fazel et al. [70] showed that mood stabilizers were significantly related to a reduced rate of violence only in patients with bipolar disorder, whereas their potential effects were not demonstrated in those with schizophrenia and related psychoses.

### Factors associated with violence perpetrated by caregivers

Caregivers who reported having another person in charge of caring were most likely prone to perpetrate violence against their ill relatives. This finding might indirectly reflect the role of caregiving burden in violence within the caregiving relationship. Indeed, having another person in charge of caring additionally to the patient diagnosed with schizophrenia results in a greater financial burden and this may explain the association found in this study. In this line, a Tunisian study found that 42.3% of caregivers of patients with schizophrenia spectrum and bipolar disorders exhibited high levels of perceived burden, and that those having (an)other individual(s) in charge had significantly greater burden [71]. A qualitative study indicated that reactions of anger and beating towards the patient were common ways of coping in caregivers of patients with schizophrenia, who also reported struggling with a heavy financial burden [62]. Feelings of burden have also been reported as linked to greater risk of caregiver violence and mistreatment in other populations, such as community elderly dependents [72,73]. It has been suggested that, in family caregiving, values of obligation toward the care of the ill relative in certain cultures (including Arab Muslim backgrounds) may have detrimental effects on caregivers’ health [74]. This sense of duty might lead certain caregivers to care for more than one relative, making demands of caregiving rapidly exhausting and overwhelming. More international research from diverse cultural groups are still needed to confirm these assumptions [75].

## Study limitations and strengths

The present study has some limitations that need to be addressed. The relatively small sample size impedes the generalization of the study results. Additionally, the investigation of violence perpetration and violence victimization was not based on psychometric measures. This was due to the lack of measures specifically aimed at assessing violence directed against, or perpetrated by caregivers of patients with mental illness, to the best of our knowledge. Moreover, as this study was cross-sectional, further longitudinal studies would be of great interest to better elucidate the causal relationships between violence manifestations and their risk factors. Qualitative or mixed-method studies would also be of great help especially in addressing violence perpetration among caregivers, due to the lack of literature data regarding this phenomenon. Even though the database is 2 years old, the findings remain highly relevant in understanding the dynamics of violence in patients with schizophrenia and their caregivers. This research provides valuable insights into the risk factors, associations and characteristics of violence, shedding light on the complexities of victimization and perpetration in schizophrenia.

## Clinical and research implications

These limitations aside, the present findings pointed to the alarming frequencies of violence victimization and violence perpetration among a Tunisian sample of caregivers of patients with schizophrenia. The most commonly reported causes were attributed to psychiatric symptoms and to the non-adherence to prescribed treatments. Having another person in charge of caring was significantly associated with violence perpetration, and having a greater sense of caregiving burden was associated with violence victimization above and beyond psychological distress in this study. Consistent with previous findings, caregiving burden appears to play a considerable role in violence occurring within the caregiver-patient relationship [3]. These findings are highly relevant because the factors identified can be addressed and violence can potentially be prevented. While taking care of patients with schizophrenia, a close attention should also be payed to their caregivers. Family interventions need to be implemented. Caregivers’ daily challenges in dealing with the patient, their sense of burden of care, and their financial difficulties should be discussed. Additionally, providing sources of stress-management and support for caregivers, improving their access to psychiatric care, to treatments and to peer support group would be help to alleviate their sense of burden. All these interventions need to be tailored to the local cultural context. It has been, for example, recommended to take into account cultural obligations toward the care of the ill when planning for interventions to reduce caregiving burden [76].

As for pharmacological interventions, even though SGAs have long been considered as the most effective drugs for treating patients with aggression and violence [77,78], trials evidence questioned their true effectiveness in reducing aggression [79–81]. More consistent and strong evidence is available to support that clozapine remains the most effective drug in violence risk reduction in patients with schizophrenia [77,82–84], independently of its antipsychotic and sedative effects [85]. Thus, clozapine is recommended in evidence-based guidelines for the treatment of aggressive and violent behavior [86–88]. However, it remains largely underutilized in Tunisia because of the tedious and costly monitoring required, which is not often possible in Tunisian settings where access to care is already strained. Further longitudinal studies would permit the identification of long-term predictors of violence victimization and violence perpetration among caregivers, and inform more effective prevention efforts.

## Conclusion

Although preliminary and based on cross-sectional data and a relatively small sample size, our findings draw attention to the high prevalence of both violence perpetration and victimization within the caregiver-patient relationship in schizophrenia in the Tunisian context. Symptoms of illness and refusal to adhere to treatment were reported as most common causes of violence victimization by caregivers. Findings also identified caregivers’ burden and having another person in charge of caring as factors significantly associated with occurrence of any violence victimization and violence perpetration, respectively. These factors are potentially malleable, and may be helpful in targeting an at-risk population and developing appropriate prevention strategies.

## Supporting information

S1 Data STROBE-checklist-v4-combined-PlosMedicine. (DOCX)
